# The risk of pet animals in spreading severe acute respiratory syndrome coronavirus 2 (SARS‐CoV‐2) and public health importance: An updated review

**DOI:** 10.1002/vms3.1320

**Published:** 2023-12-08

**Authors:** Sina Salajegheh Tazerji, Rasha Gharieb, Mohammadreza Manouchehri Ardestani, Baharak Akhtardanesh, Farrokhreza Kabir, Bita Vazir, Phelipe Magalhães Duarte, Niloufar Saberi, Ehsan Khaksar, Sadegh Haerian, Mohamed Fawzy

**Affiliations:** ^1^ Department of Clinical Science, Faculty of Veterinary Medicine, Science and Research Branch Islamic Azad University Tehran Iran; ^2^ Young Researchers and Elites Club, Science and Research Branch Islamic Azad University Tehran Iran; ^3^ Department of Zoonoses, Faculty of Veterinary Medicine Zagazig University Zagazig Egypt; ^4^ Emergency and Critical Section Arshid Veterinary Center Tehran Iran; ^5^ Department of Clinical Science, Faculty of Veterinary Medicine Shahid Bahonar University Kerman Iran; ^6^ Department of Basic Science, Faculty of Veterinary Medicine, Science and Research Branch Islamic Azad University Tehran Iran; ^7^ Postgraduate Program in Animal Bioscience Federal Rural University of Pernambuco (UFRPE) Recife Pernambuco Brazil; ^8^ Department of Clinical Science, Faculty of Veterinary Medicine, Garmsar Branch Islamic Azad University Garmsar Iran; ^9^ Department of Clinical Science, Faculty of Veterinary Medicine, Karaj Branch Islamic Azad University Karaj Iran; ^10^ Department of Virology, Faculty of Veterinary Medicine Suez Canal University Ismailia Egypt

**Keywords:** cats, dogs, one health, SARS‐CoV‐2, Zoonoses

## Abstract

Since the outbreak of SARS‐CoV‐2 was first identified in 2019, it has been reported that the virus could infect a variety of animals either naturally or experimentally. This review discusses the occurrence SARS‐CoV‐2 in dogs and cats and the role of these animals in transmitting coronavirus disease 2019 (COVID‐19) to their owners. The data were collected from epidemiological studies and case reports that focused on studying the occurrence of SARS‐CoV‐2 in pet animals and their owners. Epidemiological studies and case reports indicate that dogs and cats are infected with SARS‐CoV‐2 either naturally or experimentally; however, the global number of naturally infected animals is far lower than the number of people who have COVID‐19. These studies demonstrate that pet animals acquire the infection from direct contact with COVID‐19‐infected owners. Currently, there are no studies reporting that dogs and cats can transmit SARS‐CoV‐2 to other animals and humans, under natural conditions. The emergence of SARS‐CoV‐2 infection in companion animals (dogs and cats) in different countries worldwide raises concerns that pets are at higher risk for spreading and transmitting SARS‐CoV‐2 to humans and other animals, which poses a hazard to the public health. Therefore, investigating the role of dogs and cats in the transmission and epidemiology of SARS‐CoV‐2 will help us to design and implement appropriate preventive measures against the further transmission of SARS‐CoV‐2.

## Graphical abstract

SARS‐CoV‐2 can be transmitted to dogs and cats through close contact with coronavirus disease 2019 (COVID‐19) infected owners.

COVID‐19‐infected owners should limit close contact with their pets and follow hygienic measures.

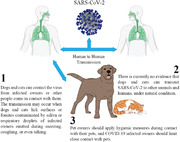

## 1 INTRODUCTION

SARS‐CoV‐2 is a newly emerged *Coronavirus* responsible for the current coronavirus disease 2019 (COVID‐19), which causes respiratory infections in humans worldwide. It is an enveloped, positive‐sense single‐stranded RNA virus that belongs to family *Coronaviridae*, genus *Betacoronavirus* (Chen et al., 2020b). The virus was initially discovered in the Wuhan Province, China in late 2019 (Bogoch et al., 2020; Zhou et al., 2020). Phylogenetic studies and genomic analysis of SARS‐CoV‐2 and its closest relatives showed that SARS‐CoV‐2, which is the causal agent of COVID‐19, has 96.2% overall genome sequence identity with bat CoV RaTG13 (Zhou et al., 2020). SARS‐CoV‐2 could be potentially transmitted to a human population, quickly adapted to humans, and human‐to‐human transmission becomes an almost immediate source of subsequent infection through direct contact and aerosol droplets (Li et al., 2020). Importantly, several studies worldwide reported that dogs and cats can be naturally (Bosco‐Lauth et al., 2020; Elaswad et al, 2020; Fritz et al., 2020; Gaudreault et al., 2020; Halfmann et al., 2020; Musso et al., 2020; Newman et al., 2020; Salajegheh Tazerji et al., 2020; Zhang et al., 2020) and experimentally (Shi et al., 2020) infected with SARS‐CoV‐2. The close association between humans and their pets raises concerns about the potential risks of transmission of SARS‐CoV‐2 from COVID‐19‐infected owners to their pets (reverse zoonosis) and the potential role of infected animals in spreading the disease (Leroy et al, 2020). Previous studies in Hong Kong (Sit et al., 2020), Brazil (Calvet et al., 2021), Latin America (Carlos et al., 2021), Italy (Patterson et al., 2020), France (Sailleau et al., 2020) and Spain (Segalés et al., 2020) demonstrated that SARS‐CoV‐2 infection is mostly detected in dogs and cats residing in households with SARS‐CoV‐2 infected owners, suggesting that human to pet transmission may have occurred. Currently, The World Health Organization (WHO), the World Organization for Animal Health, the World Small Animal Veterinary Association, as well as other human and animal health organizations indicate that there is no scientific evidence that companion animals like dogs and cats can transmit SARS‐CoV‐2 to humans. The animal origin of the SARS‐CoV‐2 led to a debate about the possible transmission of the disease to humans through animals. The investigation of the reservoir and intermediate hosts for SARS‐CoV‐2 can help in understanding the epidemiology and dynamics of the COVID‐19 pandemics, which raises the alarm that infected animals may become potential transmitters to humans. Dogs and cats are the most common species kept as household pets, thus this review discusses susceptibility of dogs and cats to infection with SARS‐CoV‐2 with a particular focus on possible transmission routes and the appropriate preventive and control measures based on One‐Health approaches.

## 2 CORONAVIRUSES PATHOTYPES IN DOGS AND CATS

Coronaviruses (CoVs) are enveloped, single‐stranded and positive‐sense RNA viruses that belong to the subfamily orthocoronavirinae, family *coronaviridae*, order *Nidovirales* and subfamily Coronavirinae, which includes four genera: Alpha‐, *Beta‐*, *Gamma*‐ and *Delta* CoVs. CoVs are identified in several species of mammals and humans (Cohen et al, 2013).

### 2.1 Coronaviruses in dogs

Dogs are infected with two types of CoVs: Canine CoVs (CCoVs), which belonged to genus *Alpha* CoVs, and Canine respiratory CoVs (CRCoVs), which belonged to genus *Beta* CoVs. CCoVs are classified into two genotypes (I and II) based on variations in spike (S) protein amino acid sequences (Licitra et al, 2014). Both CCoVs genotypes (I and II) are restricted to the gastrointestinal tract of infected dogs and known to cause enteric infections. The symptoms are generally mild and self‐limiting in the absence of co‐infecting pathogens such as parvovirus; however, gastroenteritis may occur (Buonavoglia et al., 2006; Erles et al, 2003). Also, there is a highly virulent biotype of CCoVs, which belonged to CCoV‐IIa genetic cluster and was designated as pantropic CCoV due to its systemic distribution in internal organs. Young puppies are more likely to experience the more severe pantropic CCoV symptoms characterized by fever, lethargy, haemorrhagic diarrhoea, severe lymphopenia and neurological signs followed by death (Buonavoglia et al., 2006; Decaro et al., 2007). In wild carnivorous, CCoVs were detected in wolves (*Canis lupus*), red foxes (*Vulpes vulpes*), Eurasian otters (*Lutra lutra*) and common genets (*Genetta genetta*). CRCoV infections in dogs are associated with mild respiratory signs, though severe clinical signs have been reported (Erles et al., 2003).

### 2.2 Coronaviruses in cats

Feline CoVs (FCoVs), which belong to genus *Alpha* CoVs are classified into two antigenically distinct serotypes (FCoVs I and II) and known to infect domestic and wild felids (Jaimes et al, 2020; Pedersen, 2014). Based on pathobiology and virulence, FCoVs can have either a low‐virulent enteric pathotype (feline enteric coronavirus; FECV) or a high‐virulent pathotype (feline infectious peritonitis virus; FIPV). FECV‐infected cats usually exhibit mild or subclinical symptoms during the initial viral infection but may occasionally experience transient episodes of diarrhoea and/or mild upper respiratory signs from which they recover spontaneously (Brown et al, 2009). However, FECV has the potential to be converted to a highly virulent circulating form (FIPV) if the virus mutates during replication in macrophages. FIPV that has a higher propensity to cause severe to lethal feline infectious peritonitis (FIP) (Brown et al., 2009). FIP is often observed in young cats, which has two progressive and ultimately fatal forms: The wet/effusive form associated with the accumulation of a characteristic viscous yellow fluid in body cavities and the dry/non‐effusive form with pyogranulomatous lesions affecting several organs (Kipar & Meli, 2014; Pedersen, 2014). FIP is complicated by antibody‐dependent enhancement of infection, where sub‐neutralizing antibodies can recognize the virus spike protein, enhancing macrophage infection and leading to more rapid disease progression (Takano et al, 2008). CCoVs can be transmitted easily to humans through droplets, whereas transmission of FCoVs to humans is highly limited (Jaimes & Whittaker, 2018).

## 3 OCCURRENCE OF SARS‐COV‐2 IN DOGS AND CATS

SARS‐CoV‐2 initially emerged in late 2019 in Wuhan City, China, with a symptomatic infection or severe pneumonia in individuals that resulted in mortality and was termed COVID‐19 by WHO (Tan et al., 2020; Zhu et al., 2020). Several studies investigated the occurrence of SARS‐CoV‐2 in dogs and cats (Table 1). SARS‐CoV‐2 was identified in dogs and cats living in the same households with COVID‐19‐infected owners (Table 1). However, there is no substantial evidence to date that dogs and cats can transmit SARS‐CoV‐2 to humans. The genetic relationship between SARS‐CoV‐2 recovered from infected cats, dogs and their owners residing in the same households was determined in previous studies (Table 1). A high genetic similarity with 99.8% (Barrs et al., 2020) and 99.4% (Carlos et al., 2021) was reported between SARS‐CoV‐2 genomic sequences in household cats and their owners. Furthermore, the genomic sequences of SARS‐CoV‐2 from dogs in Hong Kong, China shared a high genetic similarity (100%) with SARS‐CoV‐2 from infected owners (Sit et al., 2020).

**TABLE 1:** Reports of SARS‐CoV‐2 natural infection in dogs and cats worldwide.

Species	Country	Clinical signs	Sample	Tests	Positive/total animals (%)	Dogs/Cats are living in the same household with COVID‐19‐infected owners	Identity between the genomic sequences of SARS‐CoV‐2 in pets and their owners	References
**Dog**	Hong Kong, China	No	Oral and faecal swab Serum samples	RT‐qPCR PRN	1/1 (100.0)	Yes	NA	WHO (2022)
**Dog**	Hong Kong, China	No	Nasal and oral swabs Serum samples	RT‐qPCR PRN	1/1 (100.0)	Yes	NA	WHO (2022)
**Dog**	Croatia	No	Serum samples	Serological tests	2/654 (0.31)	Yes	NA	Stevanovic et al. (2021)
**Dog**	Hong Kong, China	No	Nasal, oral, rectal swabs and serum samples	RT‐qPCR PRN	2/15 (13.3)	Yes	100%	Sit et al. (2020)
**Dog**	Italy	Yes	Oropharyngeal, nasal, rectal swabs and serum samples	RT‐qPCR, PRN	6/47 (12.8)	Yes	NA	Patterson et al. (2020)
**Dog**	Wuhan, China	No	Oral, rectal swabs Serum samples	RT‐qPCR PRN Serological tests	1/9 (11.1)	Yes	NA	Chen et al. (2020a)
**Dog**	France	Yes	Nasopharyngeal, rectal swabs and serum samples	RT‐qPCR, serological tests	0/12 (0)	Yes	NA	Sailleau et al. (2020)
**Dog**	La Rioja, northern Spain	No	Oropharyngeal and rectal swabs	RT‐qPCR	0/12 (0)	Yes	NA	Ruiz‐Arrondo et al. (2021)
**Dog**	Thailand	Yes	Serum samples	Serological tests	35/2103 (1.66)	No	NA	Udom et al. (2022)
**Dog**	France	No	Serum samples	Serological tests	2/13 (15.4)	Yes	NA	Fritz et al. (2020)
**Dog**	Rio de Janeiro, Brazil	Yes	Nasopharyngeal, oropharyngeal, rectal swabs and serum samples	RT‐qPCR, PRN	9/29 (31)	Yes	NA	Calvet et al. (2021)
**Dog**	New York State, Richmond , USA	Yes	Serum samples	Serological tests	2/2 (100.0)	Yes	NA	Cima and Behravesh (2021)
**Dog**	Brazos, Texas, USA	No	Nasal/Nasopharyngeal, oral/oropharyngeal, rectal swabs and serum samples	RT‐qPCR, serological tests	1/59 (1.7)	Yes	NA	Hamer et al. (2020)
**Cat**	Wuhan, China	No	Nasopharyngeal and anal swabs, serum samples	Serological tests	3/15 (20)	Yes	NA	Zhang et al. (2020)
**Cat**	Croatia	No	Serum samples	Serological tests	1/131 (0.76)	Yes	NA	Stevanovic et al. (2021)
**Cat**	Hong Kong, China	No	Nasal, oral and rectal swabs	RT‐qPCR PRN	6/50 (12)	Yes	99.8%	Barrs et al. (2020)
**Cat**	Hong Kong/China	No	Nasal, oral and faecal Swabs	RT‐qPCR	1/17 (5.9)	Yes	NA	AVMA (2020)
**Cat**	Belgium	Yes	Vomit and faeces samples	RT‐qPCR	1/1 (100.0)	Yes	NA	WHO (2022)
**Cat**	New York State, USA	Yes	Nasal swabs	RT‐qPCR	2/2 (100.0)	Yes	NA	Stevanovic et al. (2021); WHO (2022)
**Cat**	Italy	Yes	Oropharyngeal, nasal, rectal swabs and serum samples	RT‐qPCR, PRN	1/22 (4.5)	Yes	NA	Patterson et al. (2020)
**Cat**	Italy	Yes	Nasal swabs	RT‐qPCR	1/1 (100)	ND	NA	Musso et al. (2020)
**Cat**	Spain	Yes	Nasal, rectal, lung swabs, mesenteric lymph node, nasal turbinates and serum samples	RT‐qPCR, serological tests	1/1 (100)	Yes	NA	Segalés et al. (2020)
**Cat**	Wuhan, China	No	Oral, rectal swabs Serum samples	RT‐qPCR, PRN, serological tests	2/10 (20)	Yes	NA	Chen et al. (2020a)
**Cat**	France	Yes	Nasopharyngeal, rectal swabs and serum samples	RT‐qPCR, serological tests	1/22 (4.5)	Yes	NA	Sailleau et al. (2020)
**Cat**	La Rioja, northern Spain	No	Oropharyngeal and rectal swabs	RT‐qPCR	1/8 (12.5)	Yes	NA	Ruiz‐Arrondo et al. (2021)
**Cat**	Thailand	Yes	Serum samples	Serological tests	4/1112 (0.36)	No	NA	Udom et al. (2022)
**Cat**	France	No	Serum samples	Serological tests	8/34 (23.5)	Yes	NA	Fritz et al. (2020)
**Cat**	Rio de Janeiro, Brazil	Yes	Nasopharyngeal, oropharyngeal, rectal swabs and serum samples	RT‐qPCR, PRN	4/10 (40)	Yes	NA	Calvet et al. (2021)
**Cat**	Brazos, Texas and USA	No	Nasal/nasopharyngeal, oral/oropharyngeal, rectal, conjunctival swabs and serum samples	RT‐qPCR, Serological tests	3/17 (17.6)	Yes	NA	Hamer et al. (2020)
**Cat**	Latin America	Yes	Nasopharyngeal and rectal samples	RT‐qPCR	1/1 (100)	Yes	99.4%	Carlos et al. (2021)
**Cat**	Italy	No	Oropharyngeal, nasal and rectal swabs, serum samples	RT‐qPCR, serological tests	0/99 (0)	Yes	NA	Stranieri et al. (2021)

Abbreviations: COVID‐19, coronavirus disease 2019; NA, not applicable; ND, not determined; PRN, plaque reduction neutralization; RT‐qPCR, reverse transcription quantitative polymerase chain reaction; WHO, World Health Organization.

## 4 CLINICAL PRESENTATION AND PATHOLOGICAL CHANGES CAUSED BY SARS‐COV‐2 IN PET ANIMALS

SARS‐CoV‐2 can be naturally and experimentally transmitted to dogs and cats, who may or may not develop clinical signs. SARS‐CoV‐2 clinical signs in infected dogs and cats are mild and self‐limiting with mainly respiratory and gastrointestinal manifestations including a combination of any of the following: sneezing, coughing, diarrhoea, anorexia, hyperthermia, lethargy, nasal discharge, regional lymphadenopathy, external otitis, perianal mucosa inflammation, hyperaemic spots on the tongue and bronchopneumonia (Calvet et al., 2021; Sailleau et al., 2020).

At 5 days post infection (DPI), the necropsy of experimentally infected cats with SARS‐CoV‐2 revealed moderate ulcerative, suppurative lymphoplasmacytic rhinitis in the nasal turbinates along with mild lymphoplasmacytic tracheitis and minimal alveolar histiocytosis with oedema (Bosco‐Lauth et al., 2020). The cats which were sacrificed at 28 DPI had moderate lymphoplasmacytic rhinitis with rare fibroplasia. Furthermore, cats which were sacrificed at 42 DPI showed minimal tracheitis, mild rhinitis and mild lung changes, including mild interstitial lymphocytic pneumonia with peribronchiolar and perivascular lymphocytic cuffing and alveolar histiocytosis. The lesions in the upper respiratory tract decreased in cats 42 DPI compared to early time point cats, whereas lung pathology was more evident in cats 42 DPI compared to those scarified during acute infection (Zhou et al., 2020).

Gaudreault et al. (2020) assessed the pathological changes in SARS‐CoV‐2‐experimentally infected cats during 4, 7 and 21 DPI. The respiratory tract showed various degrees of oedema, discoloration, congestion and atelectasis. Pathological changes were limited to the upper and lower airways (larynx, trachea, lobar and segmental bronchi of the lungs), which were characterized by multifocal lymphocytic and neutrophilic tracheobronchoadenitis of seromucous glands of the lamina propria and submucosa of the trachea and bronchi. Changes ranged from minimal to mild at four DPI and mild to moderate at seven DPI. The submucosal glands and associated ducts were distended, lined by attenuated epithelium, contained necrotic cell debris, and had mild to moderate numbers of lymphocytes, macrophages, plasma cells and neutrophilic infiltrations. No significant pathological changes were observed in pulmonary parenchyma of SARS‐CoV‐2‐infected cats on four and seven DPI. No significant pathological changes were observed in the respiratory tract at 21 DPI, with no detectable changes in the submucosa of trachea and bronchi.

## 5 RECEPTOR UTILIZATION BY SARS‐COV‐2 AND PET SUSCEPTIBILITY TO SARS‐COV‐2

The coronavirus genome encodes 16 major non‐structural proteins (nsp1 to nsp16), and four major structural proteins (Chen et al., 2020b; Helmy et al., 2020; Li, 2016). The spike (S) protein consists of two subunits which are involved in the attachment of the virus to the host cell, membrane fusion and entry into the cell. The S1 subunit amino acids receptor binding domain (RBD) interacts with the angiotensin‐converting enzyme 2 (ACE2) receptor, which is located on the surface of the host cell, to allow binding of the virion with the target cell (Lan et al., 2020; Shang et al., 2020). The S2 subunit promotes virion fusion with the host cell membrane. SARS‐CoV‐2 RBD would not only have affinity for human ACE2 receptors, but also for other animal species, including pets like dogs, cats and ferrets as well as farm animals like cattle, sheep and horses (Sun et al., 2020). The high genetic variability and apparent plasticity of the RBD allow CoVs to bind and subsequently adapt to ACE2 protein receptors of many animal species and increase the probability of the virus to cross species barriers (Andersen et al, 2020). Sequence analysis of the amino acid composition of the ACE2 contacting residues in different mammalian species demonstrated a high degree of similarity between human and feline ACE2 compared to other mammalian ACE2 receptors (Lan et al., 2020). Feline ACE2 has a score of 4, meaning that there were just two conservative substitutions among the 20 interacting residues that were different between feline and human ACE2. Moreover, contacting residues canine ACE2 had a relatively high degree of conservation with scores in the 5–6 range. These findings suggest that the SARS‐CoV‐2 spike is more likely to bind to feline ACE2 than canine ACE2 and this explains why cats are more susceptible to SARS‐CoV‐2 than dogs.

Experimental studies and sporadic reports indicate that dogs and cats are susceptible to SARS‐CoV‐2; however, cats are more susceptible than dogs (Bosco‐Lauth et al., 2020; Shi et al., 2020) and develop a much high neutralizing antibody that prevented reinfection following a second viral challenge (Bosco‐Lauth et al., 2020). Additionally, SARS‐CoV‐2 might be transmitted through respiratory droplets from experimentally inoculated cats to cats with no previous infection after cohoused contact (Halfmann et al., 2020; Shi et al., 2020).

## 6 PERSISTENCE AND SHEDDING OF SARS‐COV‐2 IN DOGS AND CATS

In experimentally infected dogs, SARS‐CoV‐2 RNA could persist in oropharyngeal and nasopharyngeal samples for 13 days following inoculation (Shi et al., 2020). SARS‐CoV‐2 RNA was detected in rectal swabs of experimentally inoculated cats for up to 14 days post inoculation, while post‐mortem examination clarified that tonsils, lymph node, olfactory pulps and spleen of scarified cats were positive for SARS‐CoV‐2 RNA on 21 days post inoculation with the highest RNA level in tonsils and lymph nodes (Gaudreault et al., 2020). SARS‐CoV‐2 RNA was detected in oropharyngeal and nasopharyngeal samples of naturally infected dogs for 14–31 days after the first positive result (Calvet et al., 2021). In naturally infected cats, SARS‐CoV‐2 RNA could persist in oropharyngeal and nasopharyngeal samples for 8–17 days (Barrs et al., 2020; Newman et al., 2020) and up to 25 days after the first positive result (Hamer et al., 2020).

The viral shedding was evaluated experimentally in earlier studies. Experimentally infected cats with SARS‐CoV‐2 could shed the virus for up to 5 days orally and nasally without developing clinical signs, whereas dogs do not appear to shed virus (Bosco‐Lauth et al., 2020). Nasal and rectal shedding of the virus was evaluated in experimentally inoculated cats with SARS‐CoV‐2 and other non‐infected cats cohoused with inoculated subjects (Halfmann et al., 2020). The authors reported that SARS‐CoV‐2 was detected in nasal swabs of inoculated and cohoused cats and virus shedding lasts for 4–5 days without any evidence of rectal shedding.

## 7 AUSPICIOUS MODE OF TRANSMISSION OF SARS‐COV‐2 BETWEEN PETS AND OWNERS

To date, there is no epidemiological evidence that dogs and cats could transmit SARS‐CoV‐2 to humans. However, all epidemiological studies indicate that dogs and cats can contract the virus from infected owners or other people who come in to contact with them. The transmission may occur when dogs and cats lick surfaces or fomites contaminated by saliva or respiratory droplets of infected owners emitted during sneezing, coughing or even talking (Ruiz‐Arrondo et al., 2021). Recent observations indicate that SARS‐CoV‐2 might be transmitted from owners to their pets by hand–mouth, eye conjunctiva or touching the nose with hands contaminated by saliva or respiratory droplets (Chen, 2020a). However, the transmission of the virus from infected people to their pets can be facilitated by some favourable risk factors, for example kissing, petting, licking or hugging (Leroy et al., 2020).

## 8 CROSS‐REACTIVITY BETWEEN SARS‐COV‐2 AND CANINE CORONAVIRUSES AND IMPACT OF PET OWNERSHIP ON THE COURSE OF SARS‐COV‐2

The efficiency of the antibody‐mediated pathway of the host immune system is related to the specificity of the antibody and the capability of the antibody to distinguish between similar and dissimilar antigens. However, the antibody could respond to other antigens similar to the target antigen which may result in cross‐reactivity and effective immunological protection similar to that produced against the primary target (Frank, 2020). A recent study conducted by Tilocca et al. (2020) hypothesized that an immunological cross‐reactivity between SARS‐CoV‐2 and CRCoV exists due to the high homology between the spike protein epitopes of the two taxonomically related CoVs. However, another investigation conducted by Lu et al. (2020) revealed that the nucleocapsid protein of CRCoV and expressed in *Escherichia coli* displayed antigenic cross reactivity with antisera against human CoVs. According to these findings, recurrent exposure to CCoVs may work as immune‐mobilization against SARS‐COV‐2 by stimulating the human immunological system and providing an effective response to SARS‐COV‐2. Thus, pet owners may contract a mild form of COVID‐19 and be asymptomatic as previously reported in Spain (Jurgiel et al., 2020).

## 9 RISK FACTORS ASSOCIATED WITH SARS‐COV‐2 INFECTION IN PETS

The main risk factors associated with SARS‐CoV‐2 infection in dogs and cats were close contact of pets (dogs and cats) with COVID‐19‐infected owners through sharing the bed with them as well as neutering of these animals (Calvet et al., 2021). Pets sharing a home with a COVID‐19‐infected person are at a higher risk of being infected with SARS‐CoV‐2 (Fritz et al., 2020).

## 10 PREVENTIVE AND CONTROL MEASURES

Although there is no evidence that dogs or cats can spread SARS‐CoV‐2 or transmit it to humans, the usual precautionary measures should be urgently considered part of a global control and ‘one health’ approach.

- Walk dogs on a leash, maintaining at least 6 feet from other people and animals. Avoid dog parks or public places where many people and dogs gather.
- Wash your pet paws with water and neutral soap or shampoo for animals (such as dog shampoo) before leaving the home.
- Disinfect bowls, toys and other animal care items with an Environmental Protection Agency‐registered disinfectant and rinse thoroughly with clean water afterwards.
- Pets that test positive for SARS‐CoV‐2 should be monitored and separated from persons and other animals until they recover.
- Pet owners without symptoms of COVID‐19 should practice good hygiene through washing their hands before and after interactions with animals and when handling animal food, waste or supplies.
- Pet owners and every eligible person in the household should be vaccinated.
- COVID‐19‐infected owners should restrict contact with pets and other people and avoid hugging, petting, kissing, licking, sharing food and sleeping in the same bed with pets.
- Pet owners suspected or confirmed to have COVID‐19 should avoid close contact with their pets and have another household member who is not infected with SARS‐CoV‐2 take care of them.
- If a sick pet owner with COVD‐19 must care for their pets, they should wear a mask and wash their hands before and after interacting with them.
- There is no evidence to date that pets could play a role in the transmission of SARS‐CoV‐2 to humans. Accordingly, there is no reason to remove pets from homes, even if COVID‐19 has been identified in members of the household.
- There is no scientific evidence that the vaccine used for dogs against CoVs provides cross‐protection against SARS‐CoV‐2; therefore, vaccination against CCoVs does not combat COVID‐19.

## 11 CONCLUSION

Collectively, epidemiological studies indicate that SARS‐CoV‐2 can be transmitted to dogs and cats through close contact with COVID‐19‐infected owners. However, there is currently no scientific evidence that dogs and cats can transmit SARS‐CoV‐2 to their owners and the COVID‐19 pandemic continues to be driven by human‐to‐human transmission. It is highly crucial to identify the zoonotic link of COVID‐19 and to evaluate the epidemiological involvement of pets in the transmission dynamics of SARS‐CoV‐2 to humans. Additionally, pet owners should apply hygienic measures during contact with their pets, and COVID‐19‐infected owners should limit close contact with pets.

## AUTHOR CONTRIBUTIONS

*Conceptualization; data curation; supervision; writing – original draft; reviewing and editing* : Sina Salajegheh Tazerji and Rasha Gharieb. *Formal analysis; writing – original; data curation, writing – original draft*: Mohammadreza Manouchehri Ardestani and Farrokhreza Kabir. *Data curation; formal analysis*: Baharak Akhtardanesh. *Conceptualization; data curation*: Bita Vazir. *Writing – original draft; writing – review and editing*: Phelipe Magalhães Duarte. *Writing – review and editing*: Niloufar Saberi and Sadegh Haerian. *Editing*: Ehsan Khaksar. *Supervision; writing – review and editing*: Mohamed Fawzy.

## CONFLICT OF INTEREST STATEMENT

The authors declare no conflicts of interest.

### PEER REVIEW

The peer review history for this article is available at https://publons.com/publon/10.1002/vms3.1320.

## Data Availability

The data used to support the findings of this study are available from the corresponding author upon request.
